# Evidence that the Density of Self Peptide-MHC Ligands Regulates T-Cell Receptor Signaling

**DOI:** 10.1371/journal.pone.0041466

**Published:** 2012-08-09

**Authors:** Nadia Anikeeva, Dimitry Gakamsky, Jørgen Schøller, Yuri Sykulev

**Affiliations:** 1 Department of Microbiology and Immunology and Kimmel Cancer Institute, Thomas Jefferson University, Philadelphia, Pennsylvania, United States of America; 2 Edinburgh Instruments Ltd., Livingston, United Kingdom; 3 Immudex Inc., Copenhagen, Denmark; University Paris Sud, France

## Abstract

Noncognate or self peptide-MHC (pMHC) ligands productively interact with T-cell receptor (TCR) and are always in a large access over the cognate pMHC on the surface of antigen presenting cells. We assembled soluble cognate and noncognate pMHC class I (pMHC-I) ligands at designated ratios on various scaffolds into oligomers that mimic pMHC clustering and examined how multivalency and density of the pMHCs in model clusters influences the binding to live CD8 T cells and the kinetics of TCR signaling. Our data demonstrate that the density of self pMHC-I proteins promotes their interaction with CD8 co-receptor, which plays a critical role in recognition of a small number of cognate pMHC-I ligands. This suggests that MHC clustering on live target cells could be utilized as a sensitive mechanism to regulate T cell responsiveness.

## Introduction

It has been proposed that T-cell responses are regulated not only by the number of pMHC ligands, but also by the spatial arrangement and the density of the ligands on the surface of APC or target cells [1], [2]. Although the number of cognate pMHC on target cells capable to elicit T-cell response was found to be remarkably low [3], [4], the recognition of such a small number of cognate ligands requires cooperation with noncognate or self pMHC [5]. To demonstrate directly cooperation between cognate and noncognate pMHC ligands several model systems have been tested, in which the pMHC proteins were assembled into oligomers containing cognate and noncognate pMHC [6], [7], [8], [9]. However, the results of these experiments were controversial. The discrepancy could be explained at least in part by the difference in relative positioning of pMHC molecules in these model systems, which has not been carefully evaluated. Meanwhile, the separating distances between pMHC molecules in these oligomers could regulate the cooperation between cognate and noncognate pMHC. We have previously utilized fluorescent nanoparticles quantum dots (QD) as a scaffold to assembled pMHC-I proteins with various biological activities at designated ratios and have demonstrated that cognate and noncognate pMHC ligands efficiently cooperate in the binding to CD8^+^ CTL and the induction of TCR-mediated Ca^2+^ signaling [8]. We have also found that noncognate pMHC-I/QD bind very efficiently to the T-cell surface, but do not initiate intracellular Ca^2+^ signaling [8]. Here we compared QD with two other scaffolds, Streptavidin and dextran, to vary multivalency and density of pMHC-I proteins assembled on these scaffolds and to study how these parameters influence the binding to live CD8^+^ T cells and the kinetics of TCR signaling.

## Results

### Noncognate pMHC/QD but not pMHC/tetramer Bind to the Surface of Live CD8^+^ T Cells

**Figure 1:**
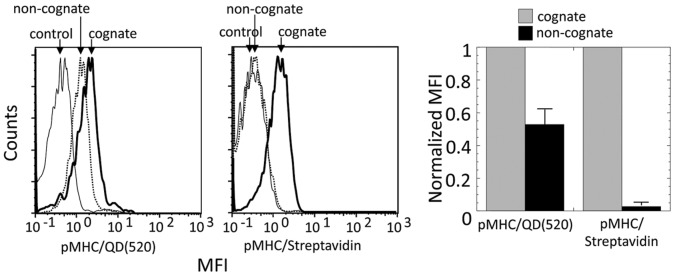
Noncognate pMHC assembled on QD but not on Streptavidin scaffold stain CD8 CTL. Left panel: Staining of 68A62 with cognate (bold solid line) and noncognate (dotted line) pMHC oligomers is shown; staining with the noncognate oligomers containing HLA-A2 with A245V mutation in nonpolymorphic domain (solid line) was used as a negative control. Right panel: Normalized MFI of 68A62 CTL incubated with cognate IV9-HLA-A2/QD(520) and IV9-HLA-A2/Streptavidin are compared with that of noncognate Tax-HLA-A2/QD(520) and Tax/Streptavidin oligomers. Data represent mean ± s.d.

To understand a unique ability of noncognate pMHC-I displayed on QD to bind vigorously to the surface of live CD8^+^ T cells [8], we first compared the binding of cognate and noncognate pMHC-I/QD with that of the same pMHC-I ligands assembled on Streptavidin scaffold into the tetramer. As expected, noncognate pMHC-I/Streptavidin did not bind to a detectable extent to the cell surface, while noncognate pMHC-I/QD did so (**Fig. 1**). The binding of noncognate pMHC-I/QD was evident at various concentrations indicating that the ability of noncognate pMHC/QD to bind to the T-cell surface was an intrinsic property of pMHC-I/QD conjugates as opposed to the tetramer (**Fig. S1**). Because QD and Streptavidin scaffolds have very similar size [10], [11], [12] but different relative orientation and proximity of pMHC-I arms, the data suggest that these parameters could be responsible for the observed distinction.

### Specific Binding of Noncognate pMHC the T-cell Surface is not a Unique Property of pMHC/QD Conjugates

**Figure 2:**
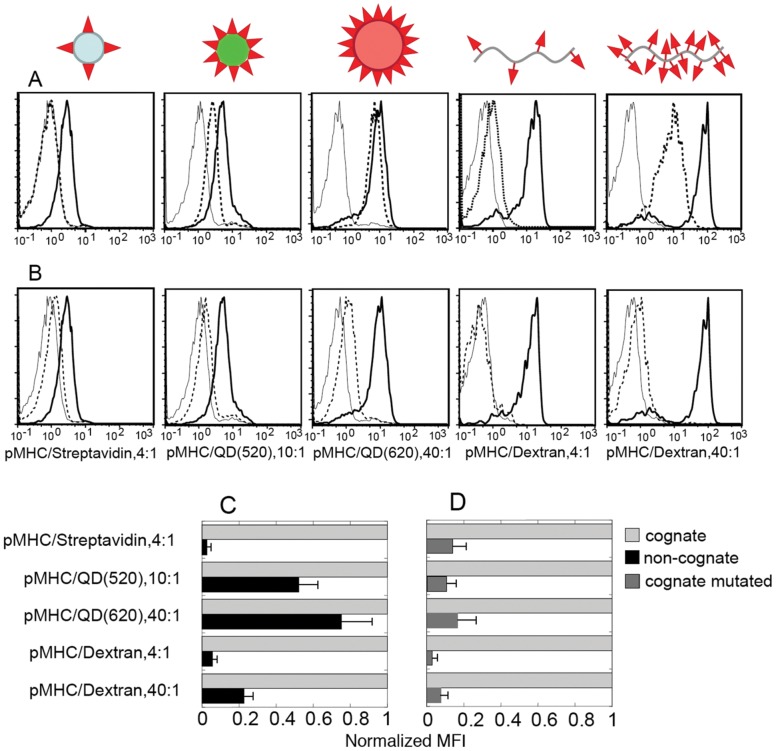
Comparison of the binding of various cognate and noncognate pMHC oligomers to CD8 CTL. A. Binding of cognate (IV9-HLA-A2, bold solid line) and noncognate (Tax-HLA-A2, dotted line) pMHC ligands assembled on different scaffolds to 68A62 CTL as established by flow cytometry. The CTL were incubated with each oligomer at 20 nM for 30 min prior to the analysis. Mutant HLA-A2(A245V) loaded with noncognate peptide (Tax) was used to produced oligomers which were utilized as negative controls (solid line). **B.** Binding of different oligomers carrying cognate peptide (IV9) in association with either intact (bold solid line) of mutated HLA-A2 (A245V) (dashed line) to 68A62 CTL was evaluated. Binding of cognate dextramers in the presence (dashed) or absence (bold solid line) of blocking anti-CD8 antibodies was examined. Other conditions are as in **A**. Data represent mean ± s.d. **C.** Normalized MFI of 68A62 bound to cognate or noncognate pMHC oligomers containing intact HLA-A2 protein. The nature of oligomers and the number of pMHC ligands per oligomer are indicated. Data represent mean ± s.d. **D.** Normalized MFI of 68A62 bound to cognate oligomers or cognate oligomers containing pMHC_mut_ with mutated HLA-A2 (A245V) protein. The tested oligomers are as in **C**.

To determine whether the binding of noncognate pMHC/QD depends on a unique property of QD scaffold, we tested the binding ability of various noncognate pMHC-I oligomers that were assembled on different scaffolds. Specifically, we used QD(520) and QD(620) that display 10 and 40 pMHC-I per dot and FITC-labeled linear dextran-based scaffold presenting either 4 or 40 pMHC-I molecules per oligomer. The orientation of pMHC-I molecules assembled on either the dextran or QD scaffolds is very different. Because of the dissimilar fluorescence of these scaffolds, relative binding of the pMHC-I oligomers was evaluated. To examine how CD8-MHC-I interactions influence the binding of cognate and noncognate pMHC-I ligands, we utilized pMHC-I oligomers containing mutation in the MHC non-polymorphic domain (MHC_mut_) or blocking anti-CD8 antibody to disrupt CD8-MHC-I interactions [8], [13]. Similar to pMHC-I/Streptavidin the binding of noncognate pMHC-I/dextran (Dextramer) containing 4 pMHC-I proteins per oligomer was barely detectable (**Fig. 2A**). However, when 40 noncognate pMHC-I molecules were assembled on the dextran scaffold of the same length, the binding of the noncognate Dextramer was clearly evident in a wide range of the Dextramer concentrations (**Fig. 2A** **and Fig. S2**). The binding was completely blocked with CD8 specific antibody as we have previously observed for various pMHC-I/QD [8], [13]. Thus, the binding of noncognate Dextramer was dependent on the pMHC-I density and was not determined by unique properties of QD scaffold. Nevertheless, relative binding of noncognate Dextramer containing 40 pMHC-I proteins was less strong than the binding of noncognate pMHC-I/QD(520) bearing 10 pMHC-I ligands per dot (**Fig. 2C**). Noncognate pMHC-I/QD(620), which have the same valency as the Dextramer, i.e., 40 pMHC per dot, bound to the cell surface even stronger than noncognate pMHC/QD(520) (**Fig 2A,C**). In contrast, the relative binding of cognate pMHC_mut_ oligomers, which was mostly determined by TCR-pMHC-I interactions, did not vary much for oligomers assembled on different scaffolds (**Fig. 2B,D**). The binding represented a small increment of the total binding of intact cognate pMHC oligomers emphasizing the importance of CD8-MHC-I interactions in the binding of all oligomers tested.

These data show that the binding of noncognate pMHC-I/oligomers to T-cell surface does not solely depend on the valency of the oligomers but is determined by other parameters such as the pMHC-I density, i.e., separating distances between neighboring pMHC-I molecules.

### Difference in the Distribution of Separating Distances between pMHC Proteins Assembled on QD and Streptavidin Scaffolds

**Figure 3:**
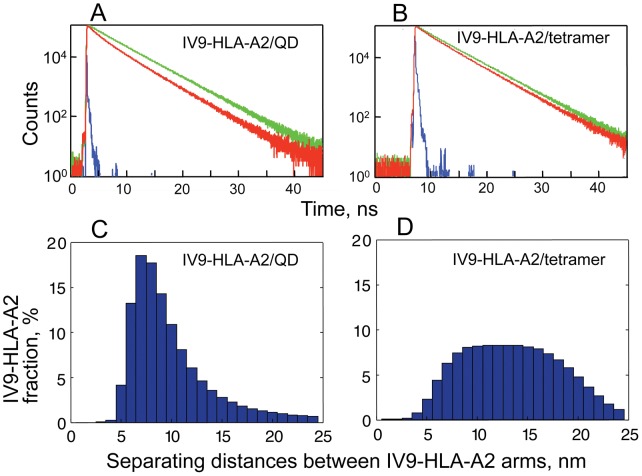
Distribution of separating distances between IV9-HLA-A2 proteins assembled on QD and Streptavidin scaffolds. Fluorescence decay of AF594-IV9 peptide bound to HLA-A2 protein (donor) in the presence or absence of AF647-IV9-HLA-A2 protein (acceptor) displayed on the surface of QD(520) (**A**) or Streptavidin scaffold (**B**). Fitting the experimental data to the model (Eqs. 1–7) yielded satisfactory fit (χ^2^<1.2) allowing to evaluate distribution of separating distances between IV9-HLA-A2 molecules displayed on QD (**C**) or Streptavidin (**D**).

To compare directly separating distances between pMHC-I proteins on the surface of QD(520) and those assembled on Streptavidin scaffold into the tetramer, we examined Förster resonance energy transfer (FRET) between AF594-IV9 (donor) and AF647-IV9 (acceptor) bound to HLA-A2 molecules (see Material and Methods for details). Natural (unquenched) fluorescence time-response of the donor was measured for the Streptavidin-based HLA-A2 tetramer in which one HLA-A2 molecule was loaded with AF594-IV9 and thee others with unlabeled IV9, as well as for the QD/IV9-HLA-A2 conjugates carrying 2 AF594-IV9-HLA-A2 and 8 unlabeled IV9-HLA-A2 (**Fig. 3A,B** **, green**). When unlabeled IV9-HLA-A2 proteins on both scaffolds were replaced with AF647-IV9-HLA-A2 molecules, the FRET was clearly evident from the changes in the lifetime of the donor fluorescence (**Fig. 3A,B** **, red**). From the best fit of the experimental data to the model described by Eqs 1-7 (see Material and Methods), we calculated distributions of separating distances between IV9-HLA-A2 molecules labeled by the donor and the acceptor on the two different scaffolds (**Fig. 3C,D**). The minimal separating distances between IV9-HLA-A2 proteins on the surface of QD were characterized by a narrow distribution with the maximum at 7.3 nm. Almost half of QD displayed IV9-HLA-A2 molecules that were separated by 5–8 nm distances. In contrast, the distribution of the minimal separating distances between IV9-HLA-A2 arms assembled on Streptavidin was significantly broader with the maximum at about 13.4 nm, i.e., 2-fold larger. Only a very small fraction (12%) of tetramers carried IV9-HLA-A2 molecules, which were separated by 5–8 nm distances. Separating distances beyond 15 nm could not be derived from FRET measurements limited by Foster radii of the fluorophores and were calculated from the model.

In accord with these data, measuring FRET efficiency between fluorescent-labeled antibodies’ Fab fragments bound to MHC-I molecules on live cells revealed that a significant fraction of the MHC-I is present in clusters [14]. In-depth analysis of these data allowed estimating distances between MHC-I molecules within clusters that were about 7–9.4 nm. These values are in a very good agreement with the minimal separating distances between IV9-HLA-A2 proteins on the surface of QD. The mean distances between cell surface MHC-I proteins calculated in the assumption on their random distribution were to be 40 nm [14]. Thus, pMHC/QD represent an adequate model of MHC-I membrane clusters.

### The Effect of pMHC Density on the TCR-mediated Signaling Kinetics

**Figure 4:**
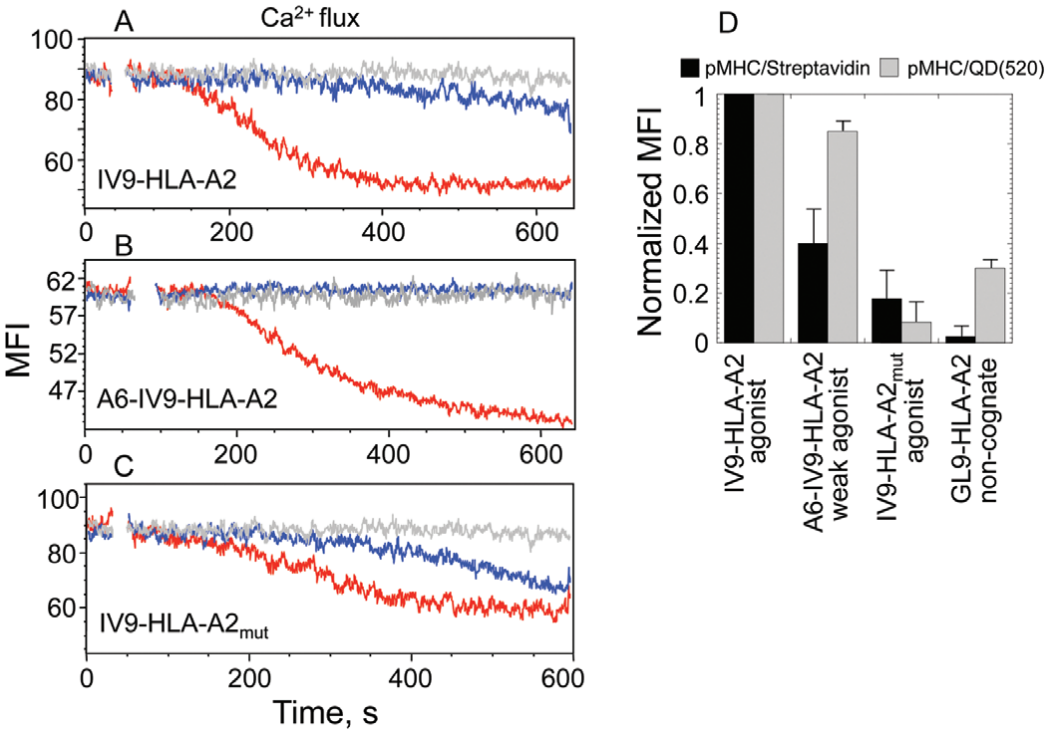
Difference in TCR-mediated signaling kinetics induced by pMHC/QD and pMHC/Streptavidin oligomers. **A**, **B**, **C.** Time-dependent changes in intracellular calcium concentration in CD8^+^68A62 CTL induced by indicated pHLA-A2 ligands assembled on either QD(520) (red) and Streptavidin (blue) scaffolds. Concentration of the probes in extracellular medium was the following: 1 nM for IV9-HLA-A2/QD(520) and IV9-HLA-A2/Streptavidin (**A**), 10 nM for A6-IV9-HLA-A2/QD(520) and A6-IV9-HLA-A2/Streptavidin (**B**), 5 nM for IV9-HLA-A2_mut_/QD(520) and IV9-HLA-A2_mut_/Streptavidin (**C**). Representative results are shown. **D.** Relative equilibrium binding of indicated pHLA-A2 ligands assembled on either QD(520) and Streptavidin scaffolds to 62A68 CD8^+^ CTL is shown. The relative amounts of cell-bound ligands were calculated from MFI measured by flow cytometry. Data represent mean ± s.d.

To test how the pMHC-I density affects TCR-mediated signaling kinetics, the binding of pHLA-A2/QD(520) and pHLA-A2/Streptavidin to 62A62 CTL and their ability to induce Ca^2+^ flux were tested. We exploited cognate strong agonist IV9-HLA-A2 and weak agonist A6-IV9-HLA-A2 ligands as well as strong agonist IV9-HLA-A2_mut_ ligand having mutation in the MHC protein disrupting CD8-HLA-A2 interactions. To evaluate kinetics of TCR signaling we measured dynamics of intracellular Ca^2+^ accumulation in a real timescale. The Ca^2+^ signaling was induced by QD(520)- and Streptavidin-based pHLA-A2 oligomers at equal concentrations of both probes and the accumulation of Ca^2+^ in the T cells was analyzed during ≈400 s, a time required for the intracellular Ca^2+^ concentration to approach maximum (**Fig. 4A–C** **and Fig S3**). The kinetics of Ca^2+^ signaling induced by IV9-HLA-A2/QD(520) was significantly more rapid as compared to that initiated by IV9-HLA-A2/Streptavidin (**Fig. 4A**). The signaling kinetics in response to IV9-HLA-A2/Streptavidin was always slower regardless of the concentration of the 2 probes (**Fig. S4**). When a weak agonist A6-IV9-HLA-A2 was used instead of a strong agonist IV9-HLA-A2 to reduce TCR-pHLA-A2 interactions, relative equilibrium binding of the tetramer was notably decreased, while the binding of A6-IV9-HLA-A2/QD(520) conjugate was only slightly lower (**Fig. 4D**). Accumulation of intracellular Ca^2+^ in 68A62 CTL was not detectable even at a higher concentration of A6-IV9-HLA-A2/Streptavidin (**Fig. 4B**). Meanwhile, A6-IV9-HLA-A2/QD(520) conjugates added to the extracellular medium at the same concentration triggered rapid kinetics of Ca^2+^ flux (**Fig. 4B**). Diminishing CD8-HLA-A2 interactions resulted in substantial decrease of relative equilibrium binding of IV9-HLA-A2_mut_/Streptavidin, and the binding of IV9-HLA-A2_mut_/QD(520) was reduced even more (**Fig. 4D**). Consequently, the difference in kinetics of Ca^2+^ flux initiated by IV9-HLA-A2_mut_/Streptavidin and IV9-HLA-A2_mut_/QD(520) was significantly decreased (**Fig. 4A and C**). Thus, stronger stimulatory potency of the cognate pMHC-I/QD was very sensitive to CD8-MHC-I interactions, while the ability of pMHC-I/Streptavidin to stimulate CTL was more dependent on the TCR-pMHC-I interactions.

**Figure 5:**
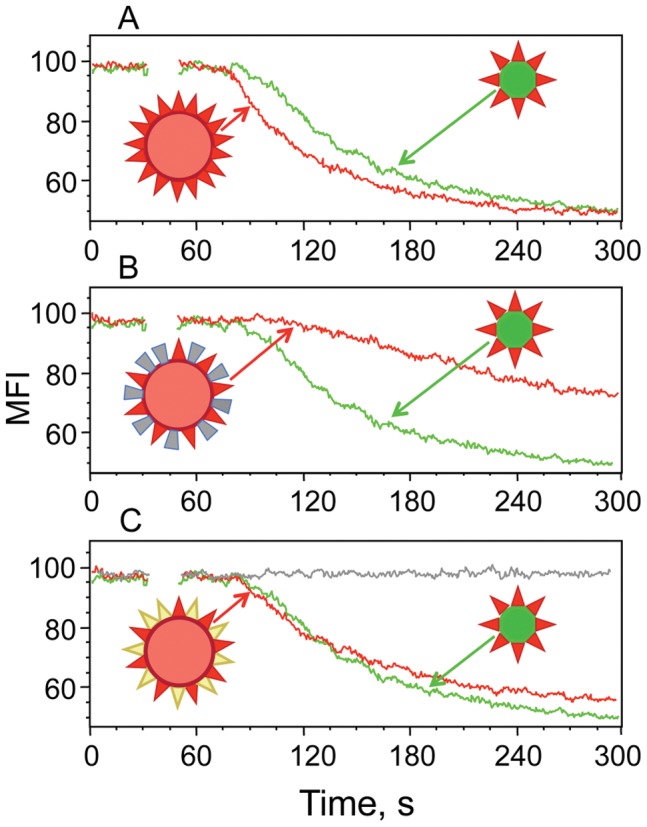
The dependence of TCR-mediated signaling kinetics upon the density of cognate and noncognate pMHC displayed on QD of different sizes. Time-dependent accumulation of intracellular Ca^2+^ in Fura Red-labeled 68A62 CTL in response to stimulation with 10 nM QD of different sizes presenting various combinations of cognate, noncognate and inactive pMHC ligands. The comparison of the stimulatory potency of (IV9-HLA-A2)_40_/QD(620) (A) or (IV9-HLA-A2)_10_(GL9-HLA-A2_mut_)_30_/QD(620) (B) or (IV9-HLA-A2)_10_(GL9-HLA-A2)_30_/QD(620) (C) versus (IV9-HLA-A2)_10_/QD(520) is shown.

To further examine the role of pMHC-I density in triggering of TCR signaling, we compared the ability of the strong agonist IV9-HLA-A2 ligands assembled on QD(520) and QD(620), which can accommodate 10 and 40 pMHC proteins per dot, respectively, to induce Ca^2+^ flux in cognate 68A62 CTL. Relative orientation and the density of IV9-HLA-A2 on QD(520) and QD(620) are very similar regardless of their size, while valency differs 4-times. **Figure 5A** shows that the increase in valency of cognate IV9-HLA-A2 ligands assembled on QD(620) as compared to QD(520) did not significantly influence the magnitude or kinetics of TCR-mediated signaling. However, QD(620) carrying 10 cognate IV9-HLA-A2 ligands per dot with all others (30 per dot) being noncognate Tax-HLA-A2_mut_ proteins (see Material and Methods) serving as irrelevant proteins were less effective in the induction of Ca^2+^ flux in 68A62 CTL (**Fig. 5B** **)**. Very similar data have been produced using different CTL clone CER43 specific for Flu-derived peptide GL9 (see Material and Methods) in association with HLA-A2 (**Fig. S5**). Thus, pMHC-I/QD having the same valency but a lower density of cognate ligands lost their capacity to elicit a rapid and robust Ca^2+^ flux in CTL.

Due to the radial/omni directional position of pMHC proteins on the surface of QD, it is not straightforward to estimate “functional valency” of pMHC-I/QD conjugates. We replaced irrelevant Tax-HLA-A2_mut_ proteins on QD(620) with noncognate Tax-HLA-A2 molecules that are not recognized by 68A62 CTL’s TCR, but interact with CD8 co-receptor. This (IV9-HLA-A2)_10_(Tax-HLA-A2)_30_/QD(620) probe had the same valency, i.e., 10 cognate pMHC per dot, while the density of HLA-A2 proteins recognizable by CD8 co-receptor was restored to 40 per dot. **Figure 5C** demonstrates that (IV9-HLA-A2)_10_(Tax-HLA-A2)_30_/QD(620) conjugates had essentially the same potency as the (IV9-HLA-A2)_40_/QD(620) and (IV9-HLA-A2)_10_/QD(520) probes. The effect was dependent on the presence of cognate pMHC-I ligands because QD(620) displaying all noncognate pMHC-I proteins, i.e., (Tax-HLA-A2)_40_/QD(620), did not induce any detectable Ca^2+^ flux (**Fig. 5C**).

These data suggest that the density of pMHC-I capable of interacting with CD8 co-receptor but not multivalency of pMHC oligomers appears to have a greater influence on the signaling kinetics.

### Cooperation between Cognate and Noncognate pMHC Depends on the pMHC Density within pMHC Oligomers

**Figure 6:**
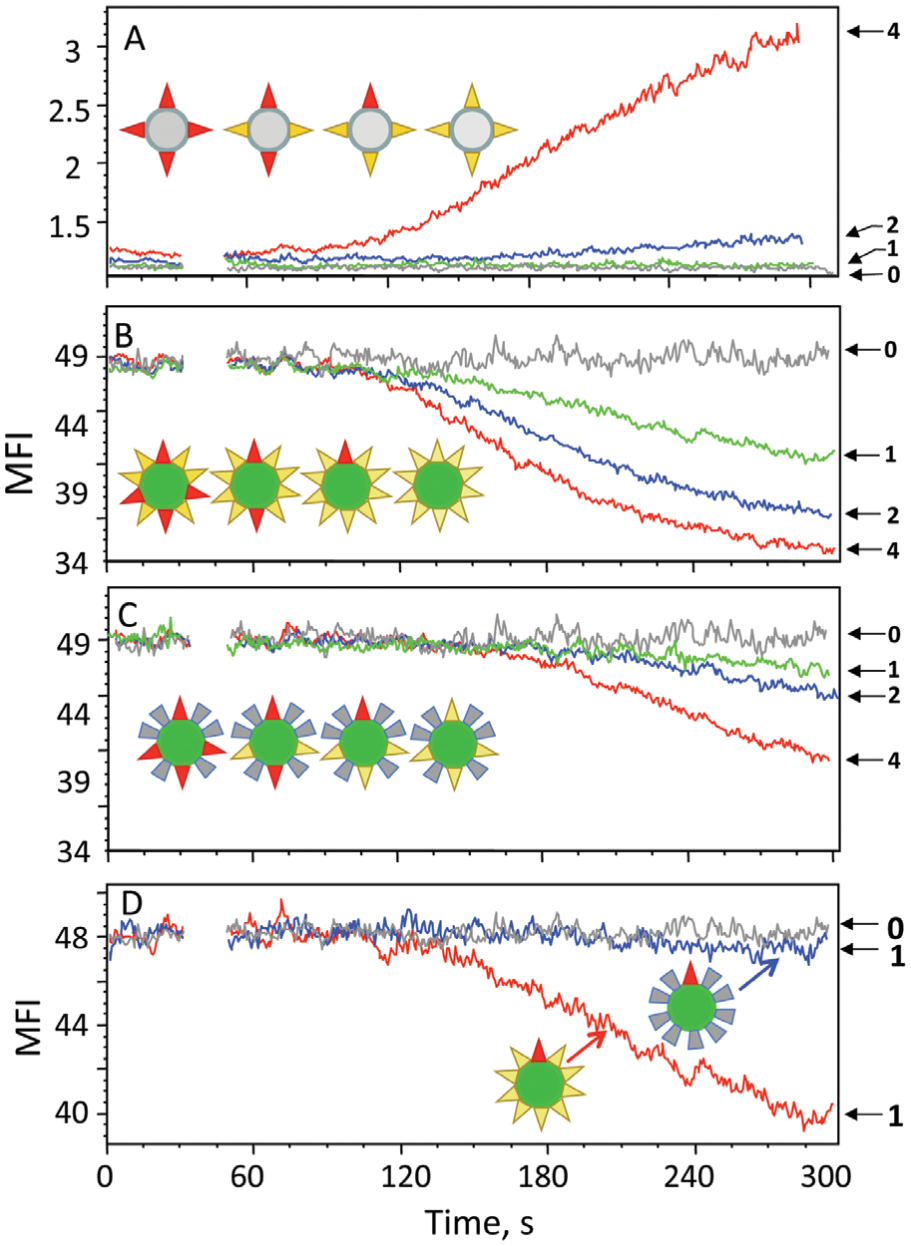
Kinetics of Ca^2+^ flux in CTL stimulated with pMHC/QD or pMHC/Streptavidin containing cognate and noncognate or inactive pMHC ligands at various ratios. A. Time-dependent Ca^2+^ accumulation in the cytoplasm of Fluo-3-labeled 68A62 CTL induced by Streptavidin-based oligomers presenting IV9-HLA-A2 (cognate, red) and Tax-HLA-A2 (noncognate, yellow) ligands at various ratios, i.e., 4∶0, 2∶2, 1∶3, 0∶4, respectively. Corresponding Ca^2+^ flux traces are designated as 4 (4∶0, red), 2 (2∶2, blue), 1(1∶3, green), 0(0∶4, grey). B, C, D. Time-dependent Ca^2+^ accumulation in the cytoplasm of Fura Red-labeled 68A62 CTL induced by QD-based oligomers presenting IV9-HLA-A2 (cognate, red), Tax-HLA-A2 (noncognate, yellow) or Tax-HLA-A2_mut_ (inactive, grey) ligands at various ratios. One series of pHLA-A2/QD conjugates being tested displayed 4, 2, 1 or 0 cognate (red) ligands per dot and 6, 8, 9 or 10 noncognate (yellow) ligands per dot keeping the total number of pHLA-A2 molecules per dot to be 10 (B). In another series of tested pHLA-A2/QD conjugates, the number of the cognate (red) ligands was the same, i.e., 4, 2, 1 or 0 per dot and the number of the noncognate (yellow) ligands per dot was 0, 2, 3, or 4, respectively, while the number of the inactive (grey) ligands and the total number of pHLA-A2 ligands per dot was kept 6 and 10, correspondingly (C). In the latter series (C), the density of noncognate Tax-HLA-A2 ligands on the conjugates was lower as compared to the conjugates of the former series (B). In the third series, QD presenting 1 cognate (red) along with either 9 noncognate (yellow) or with 9 inactive (grey) pMHC-I per dot were tested. QD presenting 10 inactive GL9-HLA-A2_mut_ proteins per dot were used as a negative control (D). Corresponding Ca^2+^ flux profiles are designated as in A.

To further investigate the role of pMHC-I density in recognition of self pMHC-I, we compared the stimulatory capacity of pMHC-I/QD(520) and pMHC-I/Streptavidin that present cognate IV9-HLA-A2 and noncognate Tax-HLA-A2 or Tax-HLA-A2_mut_ ligands at various ratios. The valency of cognate IV9-HLA-A2 proteins was varied from 1 to 4 per dot to be the same as the valency of the tetramer. Although the tetramer presenting only cognate IV9-HLA-A2 proteins (red) elicited Ca^2+^ flux in 68A62 CTL, tetramers containing cognate IV9-HLA-A2 and noncognate Tax-HLA-A2 proteins (yellow) at 2∶2 and 1∶3 ratios, respectively, were inactive (**Fig. 6A**). In contrast, QD(520) loaded with 4, 2 or 1 cognate IV9-HLA-A2 proteins per dot with all others being noncognate Tax-HLA-A2 proteins, i.e., 6, 8 and 9, correspondingly, induced Ca^2+^ responses in 68A62 CTL (**Fig. 6B**) that was comparable to that triggered by (GL9-HLA-A2)_10_/QD(520) conjugates (not shown). However, when noncognate ligands in these pHLA-A2/QD(520) conjugates were systematically replaced with inactive Tax-HLA-A2_mut_ proteins (grey), the cooperation between cognate and noncognate pHLA-A2 ligands was diminished (**Fig. 6C**). Importantly, (IV9-HLA-A2)_4_(Tax-HLA-A2_mut_)_6_/QD(520) conjugate behaved similarly to the tetramer carrying 4 cognate IV9-HLA-A2, and the stimulatory potency of the QD(520)-based conjugates declined as the number of Tax-HLA-A2_mut_ proteins per dot increased (**Fig. 6C**). As expected, (Tax-HLA-A2)_4_(Tax-HLA-A2_mut_)_6_/QD(520) probe was inactive (**Fig. 6C**).

In accord with previously published results [8], the above data show that presence of a single cognate pMHC per dot on average with all other pMHC-I being noncognate is sufficient to induce TCR-mediated signaling (**Fig. 6B**). This raises a question whether the observed T-cell response induced by QD displaying only one cognate pMHC-I could be due to a fraction of QD presenting more than one cognate pMHC. To address this issue, we tested 3 different pMHC oligomers assembled on QD(520), all presenting 10 pMHC-I proteins per dot. The two probes, QD(520) presenting 10 noncognate pMHC-I per dot and QD(520) that display 1 cognate and 9 irrelevant pMHC proteins (noncognate pMHC_mut_) per dot on average, were not able to elicit detectable Ca^2+^ flux in CTL (**Fig. 6D**). In contrast, QD(520) bearing 1 cognate and 9 noncognate pMHC-I molecules per dot on average, stimulated efficient Ca^2+^ flux in CTL (**Fig. 6D**). If only a fraction of the pMHC-I/QD(520) presenting more then 1 cognate pMHC was responsible for the ability to induce Ca^2+^ flux, then both probes presenting 1 cognate pMHC per dot on average would have been able to initiate Ca^2+^ response.

Overall, these data show that the ability of a few cognate pMHC-I ligands to elicit T-cell response requires cooperation with noncognate pMHC-I proteins and that the efficiency of the cooperation depends on the density of the pMHC-I ligands.

### Does Recognition of Noncognate pMHC by TCR Contribute to Cognate-noncognate pMHC Cooperation?

**Figure 7:**
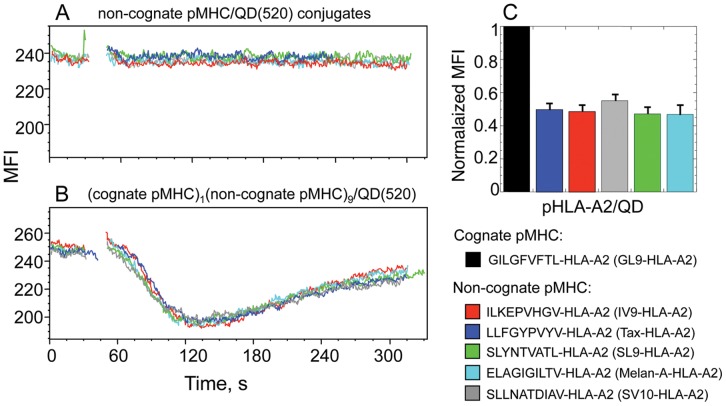
The dependence of cognate-noncognate pMHC-I cooperation on the nature of noncognate peptide. A, B. Ca^2+^ mobilization in Fura Red-labeled CER43 CTL stimulated with QD(520) that display different noncognate pMHC-I proteins (10 per dot) (**A**) or 1 cognate and various noncognate pMHC (9 per dot) (**B**) is presented. **C**. Normalized values of MFI of CER43 cells interacting with either cognate or various noncognate pMHC-I are shown. The nature of cognate and noncognate pMHC-I ligands is designated (see Material and Methods for details).

To evaluate how the nature of noncognate peptides affects cognate-noncognate pMHC cooperation, we have analyzed the ability of different noncognate pMHC-I complexes displayed along with cognate pMHC-I ligands on QDs to initiate Ca^2+^ response by CTL. We have found that all noncognate pMHC-I proteins have a very similar capacity to cooperate with cognate pMHC-I in the induction of TCR-mediated Ca^2+^ signaling (**Fig. 7B**). The difference in the binding of various noncognate pMHC-I/QD conjugates to the CTL surface was not apparent (**Fig. 7C**) presumably due to the strong contribution of CD8-MHC-I interactions that may erase small differences in the intrinsic TCR-pMHC-I affinities. Consistent with this, we have found that the binding of QD bearing various noncognate pMHC-I proteins containing mutant MHC-I(A245V) that diminishes CD8-MHC-I interactions was indistinguishable from the binding of QD displaying irrelevant pMHC-II proteins (not shown). In contrast, the contribution of noncognate pMHC-II proteins was shown to be dependent on the nature of noncognate peptide, but the role of CD4 co-receptor was less significant [7]. Binding of noncognate conjugates does not induce Ca^2+^ response of established CTL clones (**Fig. 7A**) making it difficult to study their recognition. Perhaps, analysis of other T-cell responses at various stages of differentiation and activation induced by noncognate QD/pMHC will prove to be more informative in this regard.

## Discussion

It is well established that the recognition of self pMHC is necessary for T cell development [15] and the induction of a tonic signal necessary for maintenance of peripheral T cells [16], [17]. Although these data suggest that T cells productively interact with self pMHC, the binding of self pMHC to the T-cell surface is difficult to detect due to weak interactions between self pMHC and TCR. The tetramer, a widely used pMHC oligomer, revealed relatively poor binding of noncognate pMHC-I to the T cell surface merely in special cases [18]. In contrast, our experiments described here (**Fig. 2**) and elsewhere [8], [13] clearly demonstrate that noncognate pMHC-I proteins assembled on QD effectively binds to the surface of live antigen experienced CTL. We would like to propose that the observed distinction in the binding of noncognate pMHC-I assembled on the two different scaffolds is determined by the difference in the density of the pMHC-I molecules. In fact, the analysis of separating distances between pMHC-I proteins showed that the average distance between TCR contact surfaces of pMHC-I molecules displayed on QD was 2-times shorter as compared to that found for the same pMHC-I within the tetramer (**Fig. 3**). Thus, the effect of the MHC density appears to be essential for the recognition of noncognate pMHC-I oligomers.

Despite a strong binding of noncognate pMHC/QD conjugates to the surface of CTL (**Fig. 2**), there was no detectable Ca^2+^ response induced by these conjugates even if their concentration in the extracellular medium was raised up to 100 nM (not shown). This is consistent with our previous findings [8], [13] and a prevailing view that recognition of self pMHC do not trigger conventional T-cell responses unless cognate pMHC ligands are also presented. Combining cognate and noncognate pMHC-I ligands on the surface of OD at various ratios resulted in their efficient cooperation in the induction of Ca^2+^ flux (**Fig. 5B**). However, the cooperation between the same cognate and noncognate ligands were not evident when the ligands were assembled on Streptavidin scaffold (**Fig. 5A**). Because the number of cognate ligands per oligomer in these experiments was matched, the data suggest that larger separating distances between noncognate pMHC-I molecules precluded their ability to facilitate recognition of cognate ligands. Indeed, when increasing number of noncognate pMHC-I ligands on QD scaffold was replaced with inactive pMHC-I proteins raising the pMHC-I-pMHC-I separating distances and consequently lowering the density of the noncognate pMHC-I on the QD surface, the cognate-noncognate cooperation diminished resulting in a slower kinetics of Ca^2+^ signaling (**Fig. 5C**). These data demonstrate that the density of noncognate or self pMHC-I is essential for their ability to aid recognition of cognate pMHC-I and regulates the kinetics of TCR signaling. The pMHC-I density also influences the efficiency of TCR signaling initiated by QD presenting only cognate pMHC-I. Lowering the density of cognate pMHC-I on the surface of QD(620) led to notable reduction of the amplitude and slowed down the kinetics of signaling as compared to those triggered by the same pMHC-I displayed on QD(520) at higher density (**Fig. 4F**). The impact of the pMHC-I density on the stimulatory potency was found to be stronger on the recognition of the weak agonist as opposed to the strong agonist pMHC-I ligands (**Fig. 4A,B**).

From the size of QD(520) and the dimensions of pMHC protein (including spacer) assembled on QD we have calculated the density of pMHC molecules in the pMHC/QD conjugates to be ≈7×10^3^ molecule/µm^2^. This is in a good agreement with the MHC density, i.e., 1.6−8×10^3^ molecules per µm^2^, within MHC patches (70 nm in radius) on the cell surface, each containing 25–125 MHC molecules [19]. Similar density of pMHC-I within the MHC patches suggests that noncognate pMHC-I on the surface of live APC and target cells could also cooperate with cognate pMHC-I proteins and facilitate their recognition. In fact, this was apparent from the comparison of CTL responses to target cells presenting a small number of a strong agonist pMHC-I ligand in the presence or absence of noncognate pMHC-I [20]. Therefore, the significance of the noncognate pMHC-I density that emerged from this study involving pMHC/QD conjugates appears to be relevant to that observed with live target cells.

We have previously shown that introduction of a single mutation (A245V) into non-polymorphic domain of MHC-I that diminishes CD8-MHC-I interactions or addition of blocking anti-CD8 antibody abrogate the ability of noncognate pMHC-I/QD to bind to the T-cell surface and significantly impair the cooperation between cognate and noncognate pMHC-I ligands [8], [13]. These findings have demonstrated essential role of CD8 co-receptor in recognition of noncognate pMHC-I. Here we have shown that the density of noncognate pMHC-I ligands affects their binding to CTL and the ability to cooperate with cognate pMHC-I augmenting the kinetics of TCR signaling (**Fig. 5**). We propose that the high pMHC-I density facilitates rapid on-rate of the CD8-pMHC-I interactions [21] diminishing the effect of a short half-life time of the complex and shifts the equilibrium towards the formation of CD8-MHC-I bond. Consistent with this, CTL’s encounters with target cells that present only noncognate pMHC-I still result in recruitment of CD8 co-receptor to the CTL-target cell interface indicative of efficient CD8-MHC-I interactions [22]. Raising the affinity of the CD8-MHC-I interactions leads to detectable binding of noncognate tetramer to T-cell surface [23]. Lowering pMHC density in vivo results in CD8 upregulation [17] emphasizing the role of CD8 co-receptor in recognition of self pMHC-I.

Because TCR/pMHC-I/CD8 interactions occurs between pMHC patches on target cell and TCR and CD8 co-clusters on activated T cell, efficient CD8-MHC-I rebinding allows even a single cognate pMHC protein within activating microclusters to rebind efficiently to different TCRs resulting in activation of many TCRs, a mechanism that we previously called signal spreading [8]. Efficient rebinding of CD8 co-receptor to clustered noncognate pMHC-I also promotes recruitment of p56^lck^ to activating microclusters. Elevating the density of p56^lck^ facilitates transphosphorylation contributing to TCR-mediated signal amplification. In fact, it has been recently shown that the density of Nck signaling protein plays a critical role in regulating the actin polymerization induced by TCR activation [24].

CD8 co-receptor also contributes to the recognition of cognate pMHC-I ligands [25] and kinetically promotes pMHC-I binding to TCR [26], [27]. This is in accord with previous findings showing that CD8 co-receptor facilitates TCR-pMHC interactions in a cell free system [28] and enhances the sensitivity of CTL response to target cells by one million-fold [29]. Importantly, TCR and CD8 cooperation in the recognition of cognate pMHC-I could be influenced by the nature of the peptide presented by the MHC-I proteins [30]. Indeed, the difference between “cellular” and “cell free” affinities of a TCR for diverse cognate pMHC ligands varies from 7.5 to 30 fold [31] suggesting that the CD8 contribution could depend on subtle structural changes of MHC-I proteins loaded with different peptides. While all these data suggest that TCR-pMHC-I and CD8-MHC-I interactions could influence each other, the existence of such mechanism remains to be further investigated.

Because responses of some pMHC-I-restricted T cells could be CD8-independent, allogeneic or xenogeneic responses in particular [32], [33], the cooperation between pMHC proteins during these T cell responses may not be required. It has to be noted that some MHC-I proteins could present on the cell surface as β_2_m-free heavy chains; the latter tend to form oligomeric structures [34] with a strong stimulatory potency and could cause autoimmune diseases [35]. Since β_2_m contributes to the MHC-I interactions with CD8, the recognition of β_2_m-free self pMHC proteins is likely to be CD8-independent.

Our data presented here are in accord with the results of previously published experiments in which covalently linked pMHC dimers have been exploited [6], [7], [36]. It has been shown that soluble pMHC dimers containing cognate and noncognate pMHC-I molecules linked by a rigid spacer, could specifically bind and induce activation of CD8^+^ T cells only if the spacer is short, i.e. 5–8 nm, positioning the pMHC-I molecules in a close proximity [6]. The optimal length of the spacer is very similar to the minimal separating distances between contact surfaces of pMHC-I molecules on the surface of QD that we found here (**Fig. 3**). The ability of soluble pMHC class II (pMHC-II) dimers to stimulate CD4 T cells also depends on the length of the covalent linker [36], and cognate and noncognate pMHC-II ligands linked by the short spacer show cooperation in the induction of a T cell response [7].

However, there is an important distinction between covalently linked pMHC dimers and pMHC/QD oligomers. The pMHC-I molecules attached to the QD surface have a coherent orientation and their mobility is limited as they are positioned very closely to each other, i.e. spaced by 5–8 nm minimal separating distances (**Fig. 3**). Although pMHC proteins within the dimer are linked by a rigid spacer of the same length at the C-termini [6], [36], the pMHC arms can freely rotate in solution relative to each other. At these circumstances, average separating distances between the pMHC contacting surfaces are significantly larger. Indeed, attempts to measure FRET between fluorescent-labeled peptides bound to MHC-I within the dimer have not been successful [6]. The difference in the orientation and the proximity of pMHC arms in pMHC/QD conjugates and pMHC dimers could explain why the binding of noncognate pMHC-I dimers to the CTL surface was not evident [6].

We have shown here that larger separating distances between pMHC arms within tetramer also preclude the cooperation between cognate and noncognate pMHC-I proteins (**Fig. 5A**). Similarly, when cognate and noncognate pMHC-II ligands were assembled on the Streptavidin immobilized on the glass surface, their cooperation in stimulating a response in CD4 T cells was not observed [9]. The cooperation between cognate and noncognate pMHC-II ligands was also not evident when these ligands were incorporated into glass-supported bilayers and were free to diffuse [9]. At such conditions the pMHC density in the bilayers required for cooperation could not be reached. Indeed, we have never observed cooperation between cognate and noncognate pMHC-I incorporated into the bilayers as well (Somersalo, Anikeeva, Sykulev, Dustin, unpublished data). It has also been found that at a very low density of cognate pMHC in the bilayers, conditions at which individual CD4 T cells presumably recognize a single pMHC protein, the T cells were still able to produce IL2 [9]. It is likely that very few strong pMHC agonists confined to the surface would be able to efficiently rebind to sufficient number of TCR within individual microclusters resulting from increase of the on-rate of the receptor-ligand interactions occurring between two opposing surfaces [37], [38]. The efficient pMHC rebinding to TCR could be also facilitated through restriction of the mobility of engaged pMHC molecules. It has been shown that such pMHC entrapment occurs on the surface of target cells and results in the increased sensitivity of CTL response [39].

T-cell responses to strong agonist ligands discussed above do not exclude the significance of self pMHC clustering and cooperation between agonist and self pMHC ligands. Presence of self pMHC would facilitate the kinetics of cognate pMHC identification by T cells on APC and expedite the kinetics of TCR signaling. In addition, at physiological conditions, T cells do not always face strong agonist pMHC and have to respond to a weak agonist pMHC to protect the host. We have shown that the clustering of noncognate pMHC-I proteins and their cooperation with a weak agonist pMHC-I ligand was essential to induce rapid and robust TCR signaling (**Fig. 4B**) that is linked to efficient CTL cytolytic activity [40], [41]. Thus, the major role of cognate-noncognate pMHC cooperation is to accelerate T cell hunting for the antigen and to enhance the kinetics of TCR signaling that improves the kinetics of CTL cytolytic response. This mechanism could also be important for maintaining effective responses to mutated viral epitopes that is necessary for winning the race against the virus spread [42].

In our experiments and other studies discussed here, activated T cells were utilized. It is thought that TCR on T cells could form oligomers of various sizes and the extent of TCR oligomerization could influence T cell responses [43], [44], [45], [46]. Recently it has been directly shown by near field scanning microscopy that large proportion of TCR and CD8 molecules on the surface of activated but not naïve T cells form co-clusters [47]. Thus, the interactions of pMHC-I oligomers with the surface of naïve T cells and the mechanism of initiation of TCR signaling could be very different [48].

MHC proteins also interact with other proteins on the cell surface such as ICAM-1 molecules [49] or tetraspanins [50] as well as with the cytoskeleton [51]. While formation of ICAM-1-MHC-I heteroclusters may expedite restriction of pMHC mobility on the cell membrane enhancing efficiency of antigen presentation [39], [52], tetraspanins has been shown to interact with MHC molecules presenting a particular set of peptides which may be important to increase a local concentration of these peptides [53]. Such heterotypic interactions could regulate the distribution and sorting of pMHC ligands on the cell surface that serve as a mechanism contributing to diversification of T cell responses.

## Materials and Methods

### Cells and Antibody

Human CTL clone 68A62 recognizing the ILKEPVHGV (IV9) peptide from HIV reverse transcriptase in association with HLA A2 class I MHC [54] was kindly provided by B. D. Walker. The human flu-specific CTL clone CER43 that recognizes the matrix protein peptide GILGFVFTL (GL9) [55], [56] was kindly provided by A. Lanzavecchia. Antibody specific for human CD8 co-receptor were kindly provided by Bice Perussia.

### Peptides and Soluble Peptide-MHC Complexes

HIV RT-derived peptide ILKEPVHGV (IV9) was a generous gift from H.N. Eisen. Other peptides, i.e., LLFGYPVYV (Tax) from human T lymphotropic virus type 1 [57], [58], ILKEPAHGV (A6-IV9), a synthetic variant of IV9 peptide, GILGFVFTL (GL9) from Influenza virus [55], [56], SLLNATDIAV (SV10) from HIV gp41 [59] and ELAGIGILTV (Melan-A) from melanoma [60], all were synthesized by Research Genetics, Inc. The IV9 variant ILKEPVH**C**V was stoichiometrically labeled with maleimide derivatives of either Alexa594 or Alexa647 (Invitrogen) at the Cysteine residue. Custom synthesis, purification (more than 95%) and characterization of ILKEPVHC(Alexa594)V peptide, termed AF594-IV9, and ILKEPVHC(Alexa647)V peptide, termed AF647-IV9, were performed by ProImmune Ltd. The peptide labeling at the penultimate Cysteine does not impair the peptide binding to HLA-A2 [61]. AF594-IV9 and AF647-IV9 peptides were used as a donor and an acceptor, respectively, in FRET experiments (see below).

Soluble HLA-A2 protein was expressed in S2 cells and “empty” HLA-A2 protein was purified from the culture supernatant as previously described [62], [63]. Soluble HLA-A2 molecules (3–5 mg/ml) were loaded with the unlabelled peptide of interest overnight at 23–25°C at saturating peptide concentration (10^−4^–10^−5^ M). HLA-A2 loading with fluorescent-labeled peptides was performed at 3-5 molar excess of the peptides. The mixture was incubated overnight at 37°C in the presence of protease inhibitors (Roche). Unbound peptides were removed by gel filtration on Superdex 200 HR column and purified complexes of HLA-A2 with fluorescent-labeled peptides were immediately frozen in aliquots in liquid nitrogen. Soluble His_6_-tagged pHLA-DRB1 loaded with RVEYHFLSPYVSPKESP peptide (TfR) from transferrin receptor [64] was produced and purified as previously described [40]. The TfR peptide was kindly provided by L. Stern.

### DHLA-capped Quantum Dots

Single-crystal core-shell CdSe/ZnSTOPO(trioctylphosphine oxide)-capped QDs with emission wavelength 520 nm [QD(520)] and 620 nm [QD(620)] were kindly provided by G.M. Bosak from Evident Technologies. Water-soluble aggregate-free DHLA-capped QDs were produced as previously described [40], [65].

### Assembly of pHLA-A2 Oligomers on QD, Streptavidin and Dextran Scaffolds

Assembly of IV9-HLA-A2/QD conjugates was driven by interactions of C-terminal hexahistidyl of HLA-A2 molecules with ZnS shell of QD in 10 mM sodium tetraborate buffer, 25 mM NaCl, pH 8.0 at room temperature (22–25°C). Because of the high local concentration of ZnS on the QD surface, the (His)_6_-tagged proteins binds to QD very strongly resulting in a stable conjugate formation [66]. HLA-A2 loaded with Alexa594-IV9 (donor) was first combined with QD(520) at the 2 molar excess of the protein over the QDs. HLA-A2 bearing Alexa647-IV9 (acceptor) was then added to the dots at protein-to-QD 8∶1 ratio. Thus, in each preparation two Alexa594-IV9-HLA-A2 molecules per dot served as donors while remaining Alexa647-IV9-HLA-A2 proteins were acceptor.

QD bearing cognate and noncognate pMHC ligands at various ratios were produced as previously described [8]. The total number of peptide-HLA-A2 per QD(520) was kept at 10 (**Fig. S6**), while the number of peptide-HLA-A2 per QD(620) was higher and fixed at 40. These peptide-HLA-A2/QD conjugates were used in binding experiments or to trigger TCR-mediated Ca^2+^ flux in 68A62 CTL.

Peptide-HLA-A2/tetramers containing cognate and noncognate peptide-HLA-A2 ligands at various ratios were assembled using Alexa-488-labeled or unlabeled Streptavidin from Invitrogen and Prozyme, respectively, as described previously [62], [63].

Linear pMHC oligomers assembled on dextran scaffold containing either of 4 or 40 pMHC ligands (experimental Dextramers) were produced as described previously [67], [68].

### Fluorescence Resonance Energy Transfer Measurements

The Förster resonance energy transfer (FRET) between donor (AF594-IV9-HLA-A2) and acceptor (AF647-IV9-HLA-A2) was measured from the changes of natural fluorescence time-response of the donor in the presence of the acceptor either on the surface of QD(520) or within the Streptavidin-based tetramer. Analyzing fluorescence time-response instead of fluorescence intensity in FRET measurements allows to calculate the distribution of the minimal donor-acceptor separating distances. FRET efficiency was evaluated from changes in the fluorescence time-response of non-quenched and quenched donor. The donor’s fluorescence was excited at 470 nm (8 nm bandwidth) by 100-ps pulses of a Supercontinuum laser SC450-2 (Fianium) operated at 2 MHz repetition rate. Fluorescence time-responses of the donor were measured at 630 nm (8 nm bandwidth) in a time correlated single photon counting spectrometer, LifeSpec II (Edinburgh Instruments, UK), fitted with a subtractive dispersion double monochromator, a cooled microchannel plate (MCP) photomultiplier (Hamamatsu, R3809U-50) and a Glan-Thompson polarizer and analyzer. To avoid the effects of molecular rotation on the lifetime measurements the polarizer was set vertically and the analyzer at 54.7° (the magic angle). All measurements were taken in a 3×3 mm plastic cuvette (Bio-Rad Laboratories, Hemel Hempstead, UK) in a TLC 50 temperature controlled sample holder (Quantum NortWestat, Liberty Lake, WA) at 20°C. Fluorescence time-resolved responses were evaluated either by the discrete exponential model of the FAST software (Edinburgh Instruments) or by Matlab-based software based on the Nelder-Mead minimization algorithm.

Because fluorescence time-responses of unquenched donor AF594-IV9C8-HLA-A2 confined to either QD(520) or Streptavidin deviated slightly from a single exponential pattern, a two-exponential model was found to provide a satisfactory fit of the experimental data.


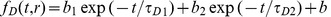
(1)

where *b*_1_ and *b*_2_ are pre-exponential coefficients; *b* is a background

The following model was used to evaluated the donor emission time-responses *I_DA_(t)* in the presence of the acceptor*:*

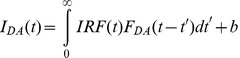
(2)where *IRF(t)* is an instrument response function and *F_DA_* is a donor’s time-response; *b* is a background. *F_DA_ (t)* is given by the following equation:


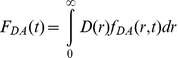
(3)

where 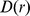 is a distribution of distances between HLA-A2 labeled with AF594-IV9 (donor) to the nearest HLA-A2 labeled with AF594-IV9 (acceptor) that is described by an asymmetrical bell-shape function:


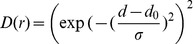
(4)

where 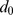 and σ are parameters describing distribution maximum and width, respectively. Asymmetry of the distribution is given by a distance-dependence of σ
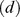



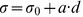
(5)

where 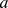 is a coefficient.

*f_DA_(d,t)* is a time-response of the donor separated by distance *d* from the acceptor.


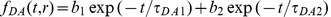
(6)

where


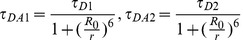
(7)

and 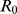 is the Förster radius (7.2 nm ) for energy transfer from AF594 to AF547 (62).

### Binding of pMHC Oligomers to Live CTL

CTL were incubated with a pMHC oligomers at various concentrations for 30 min at 4°C, washed free of unreacted conjugates, and immediately analyzed on an Epics XL-MCL flow cytometer (Beckman Coulter). To evaluate equilibrium binding of pMHC oligomers to live T cells, the washing procedure was omitted and the amount of cell bound pMHC oligomers was determined at various time points. In some experiments pMHC oligomers were assembled with a mixture of cognate and noncognate pMHC proteins at designated ratio. Noncognate pHLA-A2 proteins contained either intact HLA-A2 or HLA-A2 with a single mutation (A245V) in the nonpolymorphic domain (HLA-A2_mut_) that disrupts CD8-HLA-A2 interaction. The oligomers loaded with noncognate pHLA-A2_mut_ or pMHC class II (pHLA-DRB1) proteins were used as a control and revealed similar background staining. Loading and staining buffers contained excess of noncognate peptide (3 × 10^−4^ M) to avoid the peptide exchange. The extent of the binding of pHLA-A2 oligomers to live CTL was characterized by normalized MFI; the latter was calculated as follows: (MFI_sample_ − MFI_0_)/(MFI_cognate_ − MFI_0_), where MFI_sample_, MFI_0_, and MFI_cognate_ are measured with oligomers bearing predetermined number of tested pHLA-A2 proteins, noncognate pHLA-A2_mut_ proteins and cognate pHLA-A2 proteins, correspondingly.

### Measurements of Intracellular Ca^2+^ Flux

CTL (10^7^ per ml) were loaded with 5 µM Fura Red (Molecular Probes) or 2 µM Fluo-3 Ca^2+^ indicators in complete medium containing 4 mM probenecid and 0.02% pluronic F-127 at 37°C for 30 min. After the first wash, the cells were further incubated for 30 min at 37°C to allow de-esterification of the dye and then were washed twice and resuspended in the assay buffer (Dulbecco’s PBS containing 1 mM CaCl_2_, 0.1 mM MgCl_2_, 5 mM glucose, and 0.025% BSA) at 10^6^ per ml. After the background measurements, the pMHC oligomers of interest were promptly added to 1 ml of the CTL suspension at various concentrations, and the samples were analyzed on an Epics XL-MCL flow cytometer. The data were analyzed with FlowJo software (Tree Star, Ashland, OR).

## Supporting Information

Figure S1 **Relative equilibrium binding of noncognate pMHC/QD conjugates to CTL surface does not depend on the conjugates concentration added to the extracellular medium.** Cognate or noncognate pMHC/QD were combined with the CTL and the mixture was incubated for 30 minutes prior to flow cytometry analysis. The dependences of MFI associated with the cell surface upon concentration of cognate and noncognate conjugates added to the extracellular medium were evaluated. Actual (**A**) and normalized (**C**) values of MFI of cognate IV9-HLA-A2/QD(520) and noncognate Tax-HLA-A2/QD(520) conjugates bound to the surface of 68A62 CTL at 2 different concentrations are shown. Comparison of actual (**B**) and normalized (**D**) values of MFI of cognate GL9-HLA-A2/QD(520) and noncognate Tax-HLA-A2/QD(520) bound to the surface of CER43 CTL at indicated concentrations is presented.
(TIF)

Figure S2 **The extent of the equilibrium binding of noncognate pMHC/QD to the T-cell surface depends on the density of the pMHC ligands assembled on the dextran scaffold.** Cognate (IV9-HLA-A2) or noncognate (Tax-HLA-2) proteins were assembled on a linear fluorescent-labeled dextran scaffold to yield p-HLA-A2/dextran oligomers containing either 4 (left panels) or 40 (right panels) pHLA-A2 arms per dextran molecule of the same length. The cognate and noncognate oligomers were incubated with 68A62 CTL for 30 minutes and the amount of IV9-HLA-A2/dextran or Tax-HLA-A2/dextran associated with the surface of the CTL was determined by flow cytometry. Actual (**A** and **B**) and normalized (**C** and **D**) values of MFI at various concentrations of the tested IV9-HLA-A2/dextran or Tax-HLA-A2/dextran conjugates are shown.
(TIF)

Figure S3 **The binding kinetics of pMHC/QD and pMHC/Streptavidin oligomers to the surface of 68A62 CTL as established by flow cytometry.** The binding kinetics of various QD(520)-based conjugates (red), i.e., strong agonist IV9-HLA-A2/QD (**A**), a weak agonist A6-HLA-A2/QD (**B**) or IV9-HLA-A2_mut_/QD containing HLA-A2 mutant (A245V) (**C**), was compared with binding kinetics of Streptavidin-based conjugates (blue) containing either IV9-HLA-A2 (**A**) or A6-HLA-A2 (**B**) or IV9-HLA-A2_mut_ (**C**) to 68A62 CTL. The conjugates were added to the extracellular medium (25 nM) at time zero. Aliquots were taken at indicated time points and MFI associated with the CTL was measured by flow cytometry. The dependence of the MFI vs time is shown.
(TIF)

Figure S4 **Kinetics of TCR-mediated Ca^2+^ signaling in 68A62 CTL induced by IV9-HLA-A2/QD(520) or IV9-HLA-A2/QD/Streptavidin oligomers at various concentrations.** IV9-HLA-A2/QD(520) (**A**) or IV9-HLA-A2/QD/Streptavidin (**B**) oligomers were added to the extracellular medium of Fluo-3 labeled 68A62 CTL at indicated concentration, and changes in the fluorescent intensity of Fluo-3 as a function of time were measured by flow cytometry. The data were analyzed with FlowJo software. From the initial increase of intracellular Ca^2+^, we have determined slope for each kinetic curve. The dependence of the slope upon concentration for both oligomers is presented on panel **C**.
(TIF)

Figure S5 **The influence of the density of cognate pMHC displayed on QD of different sizes on the Ca^2+^ signaling kinetics induced in 68A62 CTL.** Kinetics of intracellular Ca^2+^ accumulation in Fluo-3 labeled 68A62 CD8^+^ CTL stimulated with cognate IV9-HLA-A2 ligands assembled on a smaller QD(520) (red trace) or a larger QD(620) (green trace) scaffolds with the same geometry. Left: the density of IV9-HLA-A2 proteins (red) on the 2 probes was similar, while the valency on the 2 probes differed by a factor of 4, i.e., 10/dot and 40/dot, respectively. Right: cognate IV9-HLA-A2 (red) on QD(620) were diluted by inactive Tax-HLA-A2_mut_ molecules (grey) to decrease the density of the cognate ligands by 4-fold, but keeping their valency similar to that on QD(520), i.e., 10/dot. Representative results are shown.
(TIF)

Figure S6 **Quantification of the number of pMHC molecules per dot in pMHC/QD conjugates.** His_6_-terminated IV9-HLA-A2 proteins were combined with QD at 10∶1 molar ratio in 10 mM sodium tetraborate buffer, 25 mM NaCl, pH 8.0 to allow self-assembly of IV9-HLA-A2/QD conjugates [8]. The conjugates were loaded on Superdex 200 HR column in the same buffer and the optical density at 280 nm was measured in the eluted fractions. The first peak of the elution profile represents pMHC/QD conjugates while the second peak corresponds to the unbound pMHC protein. At protein-to-QD ratio 10∶1, essentially all IV9-HLA-A2 molecules were bound to QD resulting in IV9-HLA-A2/QD conjugates containing 10 IV9-HLA-A2 molecules per dot (**B**). When protein-to-QD ratio was increased to 16∶1, the peak of unbound protein (**C**) corresponding to the position of soluble IV9-HLA-A2 protein (**A**) eluted in the absence of QD was substantially larger. Relative adsorption of QD and pMHC protein at 280 nm, which were determined prior to the conjugate formation, and the integrated peak area were used for quantitative analysis of SEC chromatogram. Increase of IV9-HLA-A2-to-QD ratio did not result in a notably higher number of conjugated IV9-HLA-A2 molecules (10–12.5) per dot suggesting that the IV9-HLA-A2 molecules on the surface of QD were very closely positioned to each other. The same approach was used to evaluate the number of pHLA-A2 molecules per dot in pHLA-A2/QD conjugates assemble on a larger QD(620), which were found approximately equal to 40 molecule pMHC per dot (not shown). The results of this analysis are in a good agreement with previously published data based on FRET measurements between the center of the core of QD (donor) and fluorescent-labeled IV9-HLA-A2 (acceptor) displayed on the QD surface [8].
(TIF)
