# Supplementary figures and images for: Evidence that the Density of Self Peptide-MHC Ligands Regulates T-Cell Receptor Signaling
Source: PLoS One. 2012 Aug 9;7(8):e41466. doi: 10.1371/journal.pone.0041466 (PMC3411518; doi:10.1371/journal.pone.0041466)

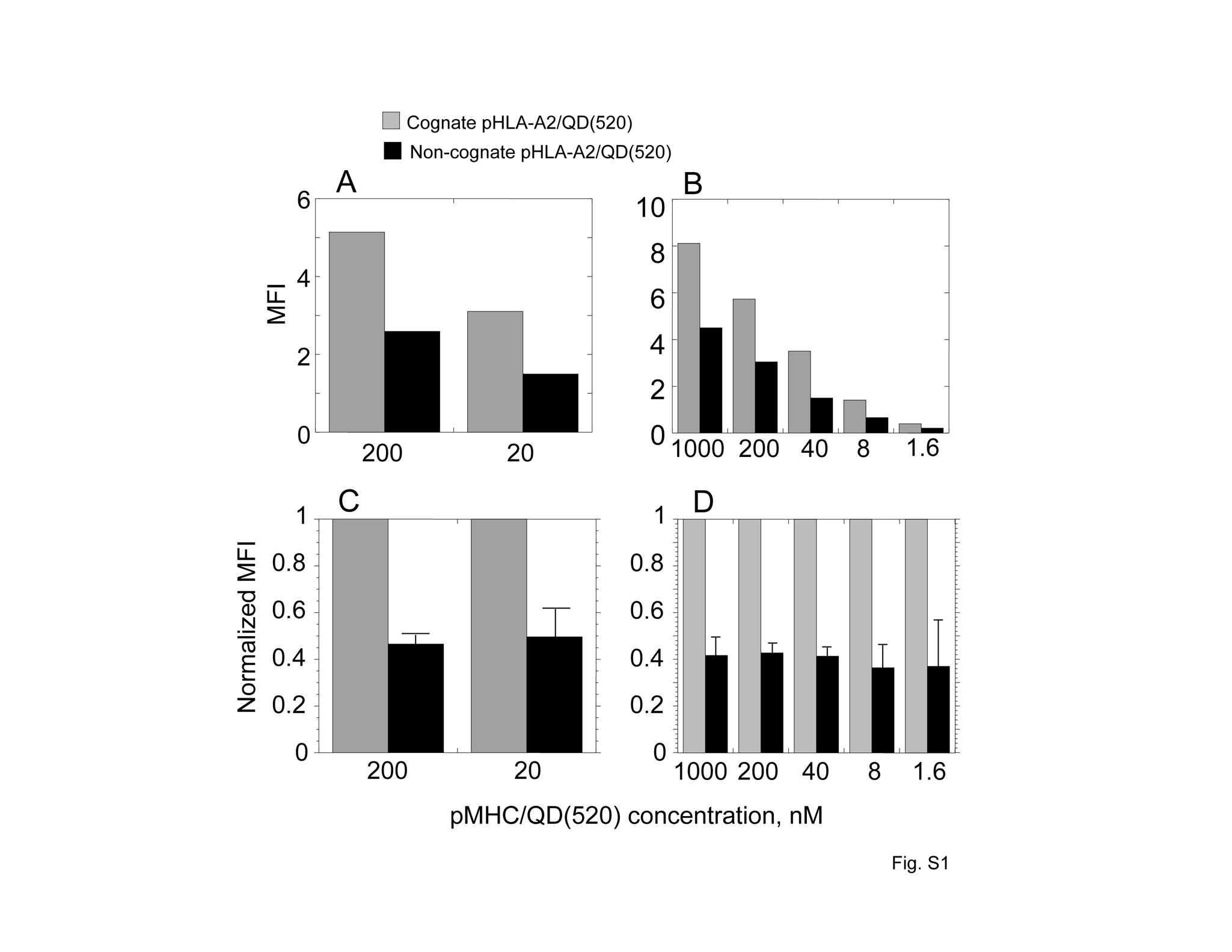

Supplement: Figure S1 — Relative equilibrium binding of noncognate pMHC/QD conjugates to CTL surface does not depend on the conjugates concentration added to the extracellular medium. Cognate or noncognate pMHC/QD were combined with the CTL and the mixture was incubated for 30 minutes prior to flow cytometry analysis. The dependences of MFI associated with the cell surface upon concentration of cognate and noncognate conjugates added to the extracellular medium were evaluated. Actual (A) and normalized (C) values of MFI of cognate IV9-HLA-A2/QD(520) and noncognate Tax-HLA-A2/QD(520) conjugates bound to the surface of 68A62 CTL at 2 different concentrations are shown. Comparison of actual (B) and normalized (D) values of MFI of cognate GL9-HLA-A2/QD(520) and noncognate Tax-HLA-A2/QD(520) bound to the surface of CER43 CTL at indicated concentrations is presented. (TIF) [file pone.0041466.s001.tif]

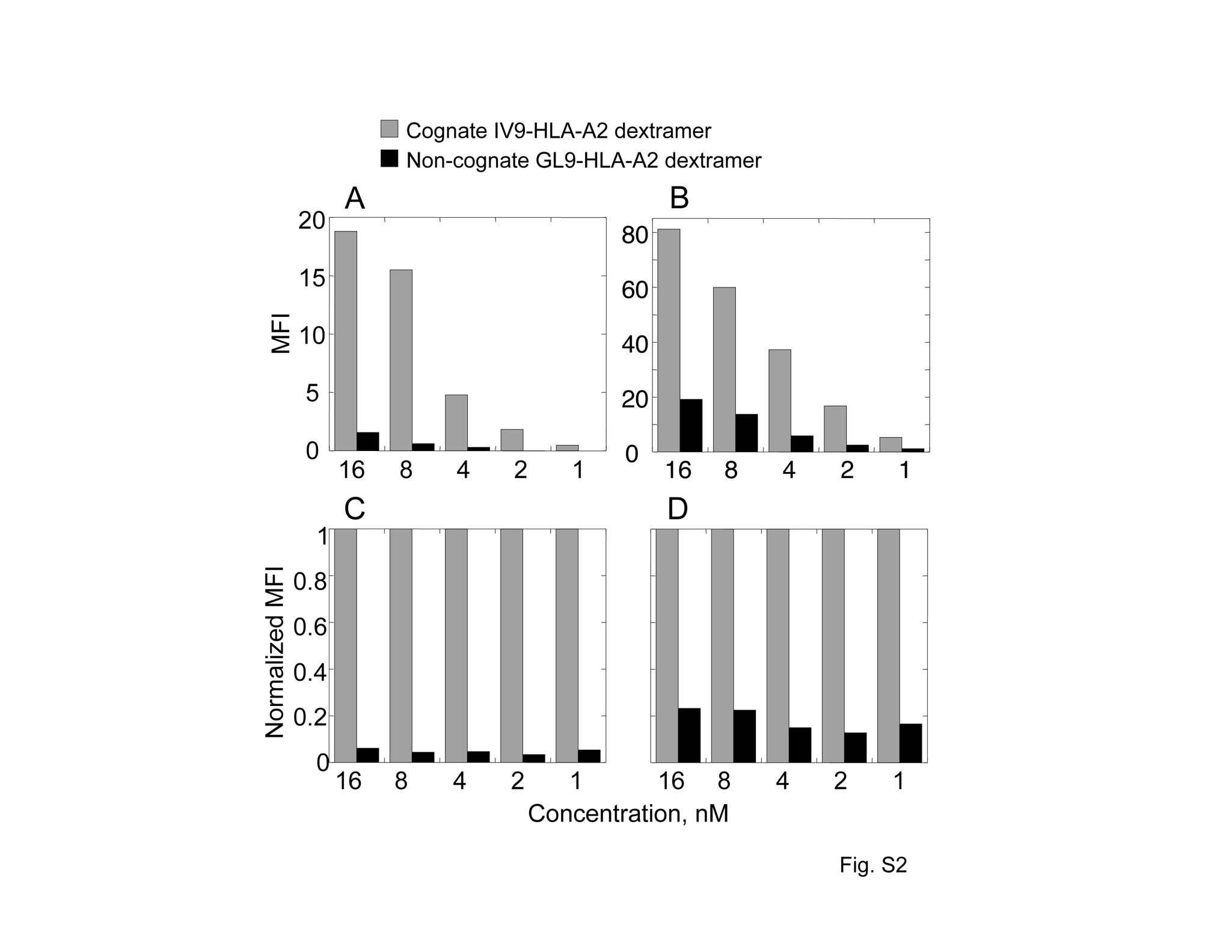

Supplement: Figure S2 — The extent of the equilibrium binding of noncognate pMHC/QD to the T-cell surface depends on the density of the pMHC ligands assembled on the dextran scaffold. Cognate (IV9-HLA-A2) or noncognate (Tax-HLA-2) proteins were assembled on a linear fluorescent-labeled dextran scaffold to yield p-HLA-A2/dextran oligomers containing either 4 (left panels) or 40 (right panels) pHLA-A2 arms per dextran molecule of the same length. The cognate and noncognate oligomers were incubated with 68A62 CTL for 30 minutes and the amount of IV9-HLA-A2/dextran or Tax-HLA-A2/dextran associated with the surface of the CTL was determined by flow cytometry. Actual (A and B) and normalized (C and D) values of MFI at various concentrations of the tested IV9-HLA-A2/dextran or Tax-HLA-A2/dextran conjugates are shown. (TIF) [file pone.0041466.s002.tif]

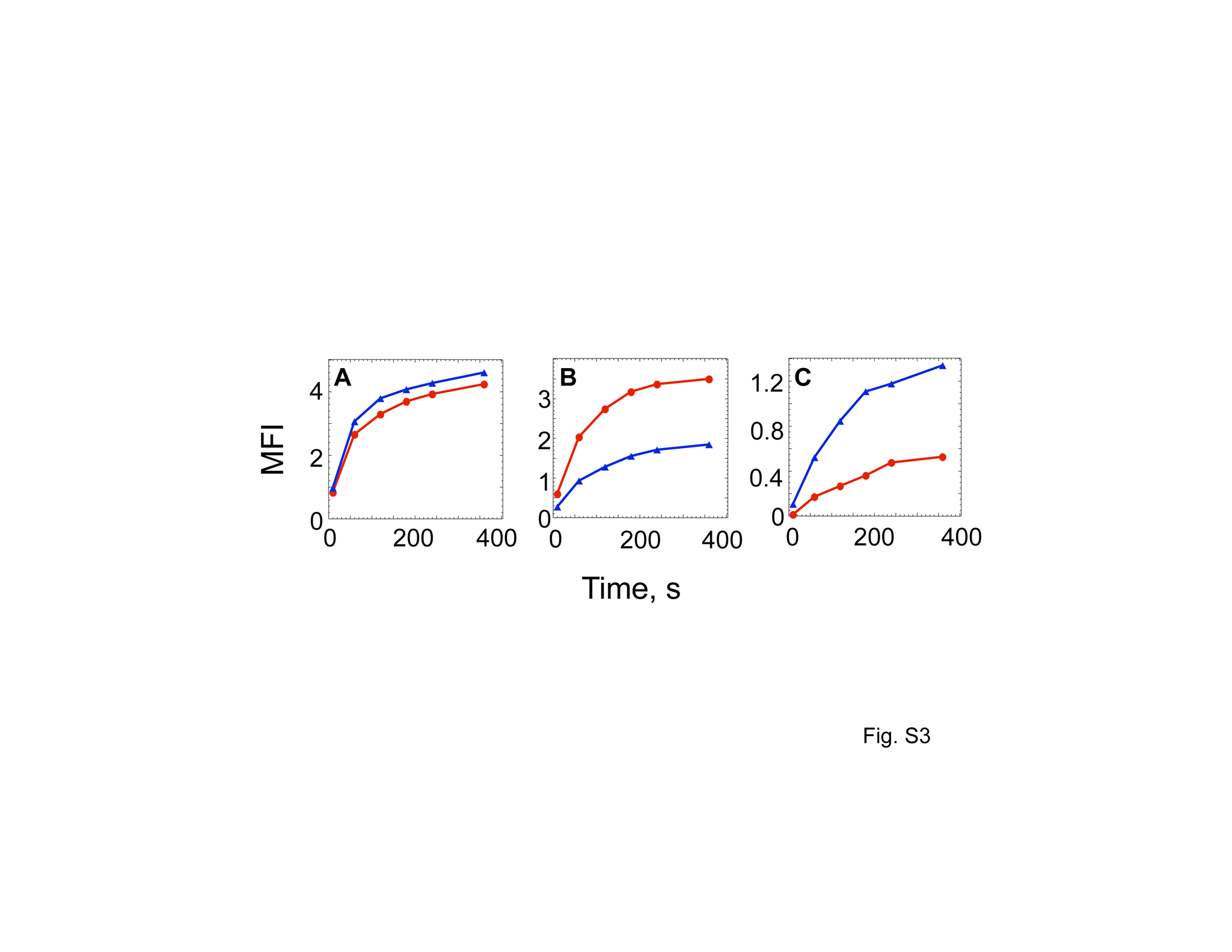

Supplement: Figure S3 — The binding kinetics of pMHC/QD and pMHC/Streptavidin oligomers to the surface of 68A62 CTL as established by flow cytometry. The binding kinetics of various QD(520)-based conjugates (red), i.e., strong agonist IV9-HLA-A2/QD (A), a weak agonist A6-HLA-A2/QD (B) or IV9-HLA-A2mut/QD containing HLA-A2 mutant (A245V) (C), was compared with binding kinetics of Streptavidin-based conjugates (blue) containing either IV9-HLA-A2 (A) or A6-HLA-A2 (B) or IV9-HLA-A2mut (C) to 68A62 CTL. The conjugates were added to the extracellular medium (25 nM) at time zero. Aliquots were taken at indicated time points and MFI associated with the CTL was measured by flow cytometry. The dependence of the MFI vs time is shown. (TIF) [file pone.0041466.s003.tif]

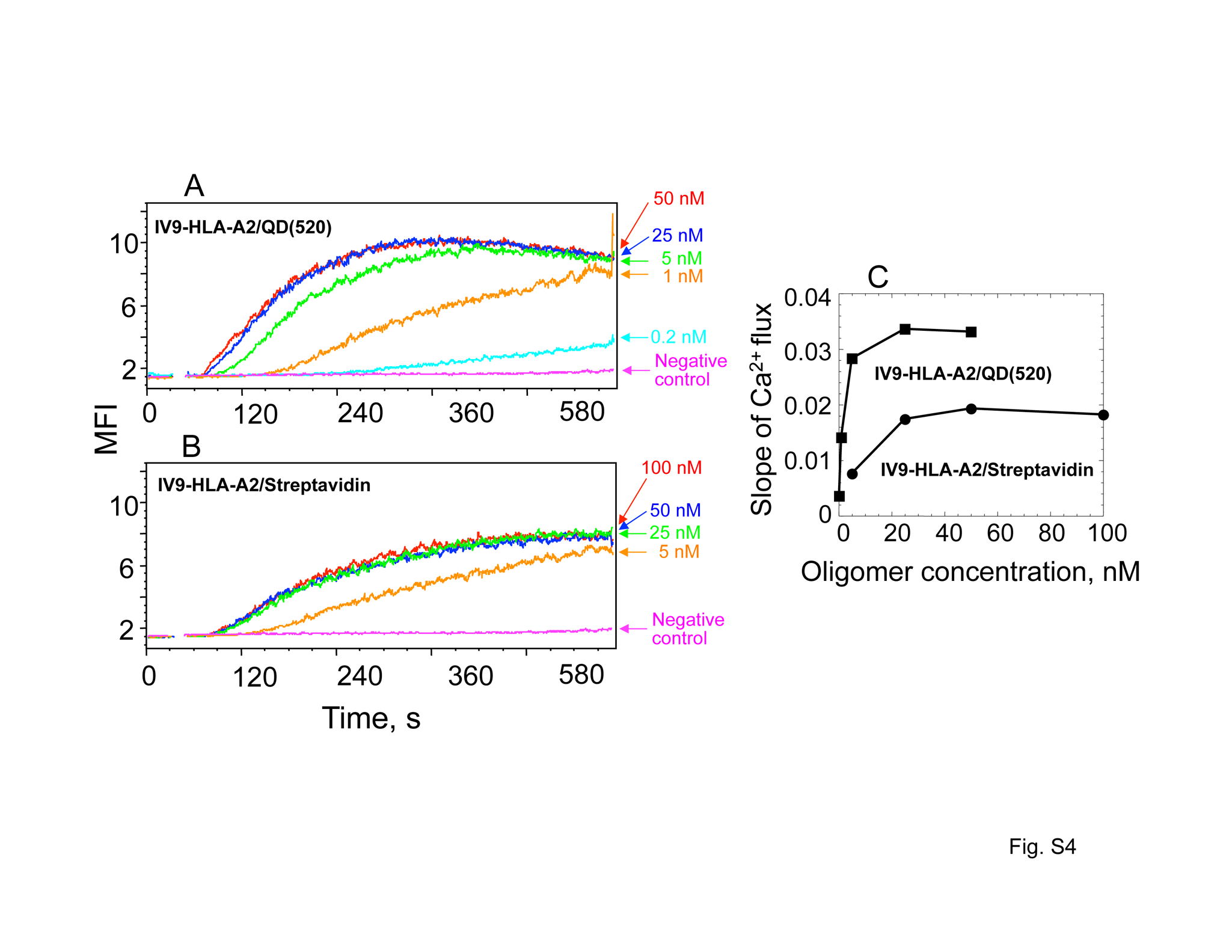

Supplement: Figure S4 — Kinetics of TCR-mediated Ca2+ signaling in 68A62 CTL induced by IV9-HLA-A2/QD(520) or IV9-HLA-A2/QD/Streptavidin oligomers at various concentrations. IV9-HLA-A2/QD(520) (A) or IV9-HLA-A2/QD/Streptavidin (B) oligomers were added to the extracellular medium of Fluo-3 labeled 68A62 CTL at indicated concentration, and changes in the fluorescent intensity of Fluo-3 as a function of time were measured by flow cytometry. The data were analyzed with FlowJo software. From the initial increase of intracellular Ca2+, we have determined slope for each kinetic curve. The dependence of the slope upon concentration for both oligomers is presented on panel C. (TIF) [file pone.0041466.s004.tif]

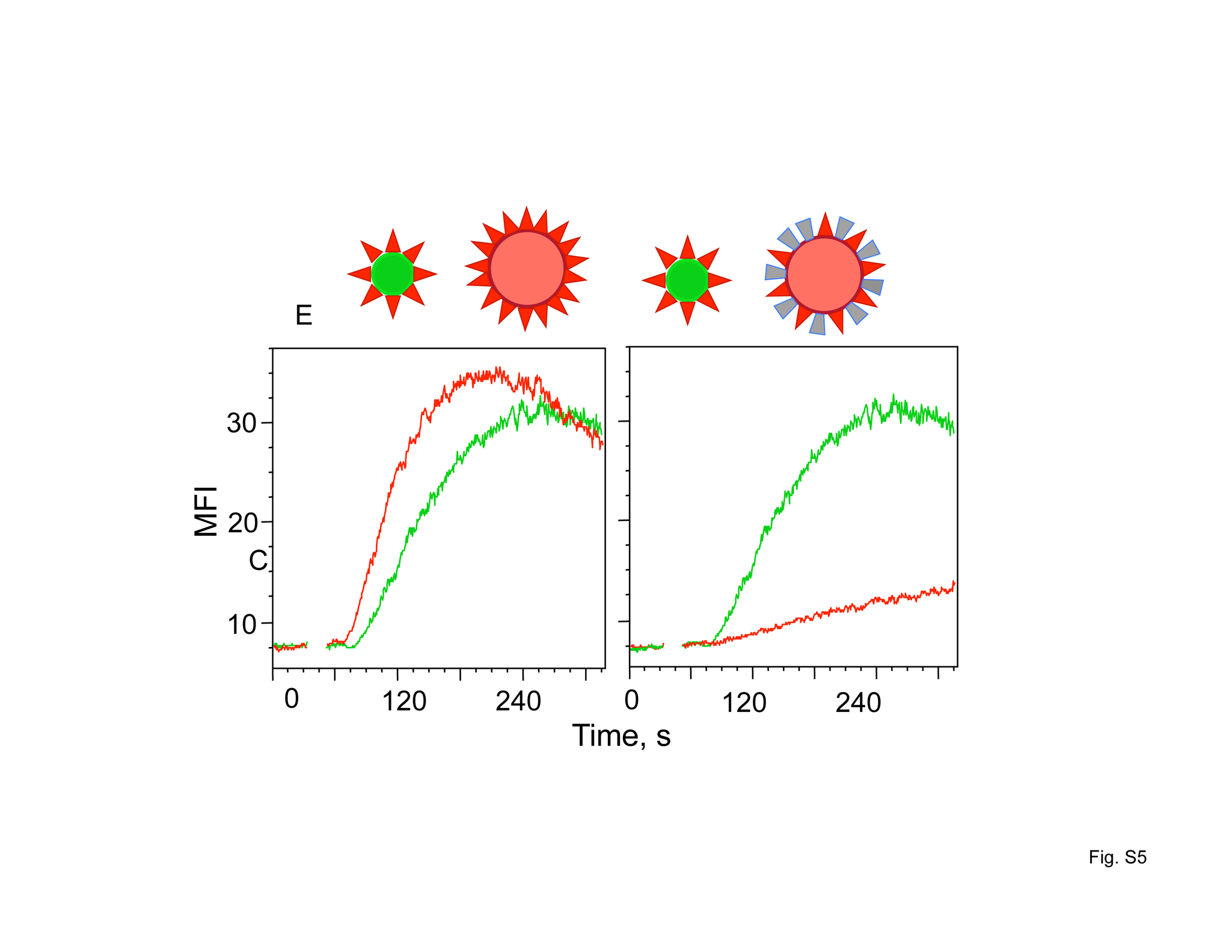

Supplement: Figure S5 — The influence of the density of cognate pMHC displayed on QD of different sizes on the Ca2+ signaling kinetics induced in 68A62 CTL. Kinetics of intracellular Ca2+ accumulation in Fluo-3 labeled 68A62 CD8+ CTL stimulated with cognate IV9-HLA-A2 ligands assembled on a smaller QD(520) (red trace) or a larger QD(620) (green trace) scaffolds with the same geometry. Left: the density of IV9-HLA-A2 proteins (red) on the 2 probes was similar, while the valency on the 2 probes differed by a factor of 4, i.e., 10/dot and 40/dot, respectively. Right: cognate IV9-HLA-A2 (red) on QD(620) were diluted by inactive Tax-HLA-A2mut molecules (grey) to decrease the density of the cognate ligands by 4-fold, but keeping their valency similar to that on QD(520), i.e., 10/dot. Representative results are shown. (TIF) [file pone.0041466.s005.tif]

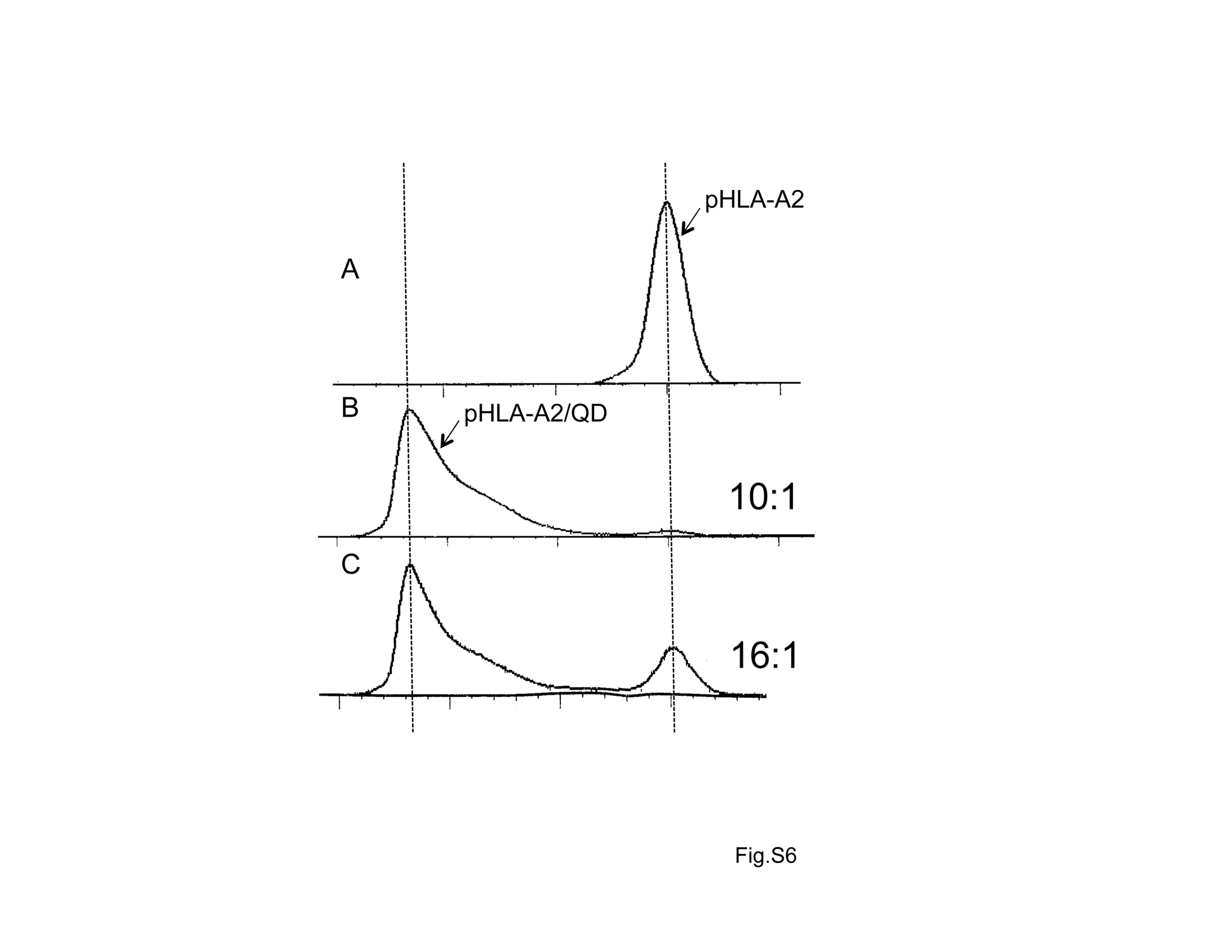

Supplement: Figure S6 — Quantification of the number of pMHC molecules per dot in pMHC/QD conjugates. His6-terminated IV9-HLA-A2 proteins were combined with QD at 10∶1 molar ratio in 10 mM sodium tetraborate buffer, 25 mM NaCl, pH 8.0 to allow self-assembly of IV9-HLA-A2/QD conjugates [8]. The conjugates were loaded on Superdex 200 HR column in the same buffer and the optical density at 280 nm was measured in the eluted fractions. The first peak of the elution profile represents pMHC/QD conjugates while the second peak corresponds to the unbound pMHC protein. At protein-to-QD ratio 10∶1, essentially all IV9-HLA-A2 molecules were bound to QD resulting in IV9-HLA-A2/QD conjugates containing 10 IV9-HLA-A2 molecules per dot (B). When protein-to-QD ratio was increased to 16∶1, the peak of unbound protein (C) corresponding to the position of soluble IV9-HLA-A2 protein (A) eluted in the absence of QD was substantially larger. Relative adsorption of QD and pMHC protein at 280 nm, which were determined prior to the conjugate formation, and the integrated peak area were used for quantitative analysis of SEC chromatogram. Increase of IV9-HLA-A2-to-QD ratio did not result in a notably higher number of conjugated IV9-HLA-A2 molecules (10–12.5) per dot suggesting that the IV9-HLA-A2 molecules on the surface of QD were very closely positioned to each other. The same approach was used to evaluate the number of pHLA-A2 molecules per dot in pHLA-A2/QD conjugates assemble on a larger QD(620), which were found approximately equal to 40 molecule pMHC per dot (not shown). The results of this analysis are in a good agreement with previously published data based on FRET measurements between the center of the core of QD (donor) and fluorescent-labeled IV9-HLA-A2 (acceptor) displayed on the QD surface [8]. (TIF) [file pone.0041466.s006.tif]
